# EG-TransUNet: a transformer-based U-Net with enhanced and guided models for biomedical image segmentation

**DOI:** 10.1186/s12859-023-05196-1

**Published:** 2023-03-07

**Authors:** Shaoming Pan, Xin Liu, Ningdi Xie, Yanwen Chong

**Affiliations:** The State Key Laboratory of Information Engineering in Surveying, Mapping, and Remote Sensing, Wuhan University, Wuhan, China

**Keywords:** Medical image segmentation, Transformer, Self-attention, Progressive enhancement module, Channel spatial attention, Semantic guidance attention

## Abstract

Although various methods based on convolutional neural networks have improved the performance of biomedical image segmentation to meet the precision requirements of medical imaging segmentation task, medical image segmentation methods based on deep learning still need to solve the following problems: (1) Difficulty in extracting the discriminative feature of the lesion region in medical images during the encoding process due to variable sizes and shapes; (2) difficulty in fusing spatial and semantic information of the lesion region effectively during the decoding process due to redundant information and the semantic gap. In this paper, we used the attention-based Transformer during the encoder and decoder stages to improve feature discrimination at the level of spatial detail and semantic location by its multihead-based self-attention. In conclusion, we propose an architecture called EG-TransUNet, including three modules improved by a transformer: progressive enhancement module, channel spatial attention, and semantic guidance attention. The proposed EG-TransUNet architecture allowed us to capture object variabilities with improved results on different biomedical datasets. EG-TransUNet outperformed other methods on two popular colonoscopy datasets (Kvasir-SEG and CVC-ClinicDB) by achieving 93.44% and 95.26% on mDice. Extensive experiments and visualization results demonstrate that our method advances the performance on five medical segmentation datasets with better generalization ability.

## Introduction

With the help of medical imaging technology, physicians can now understand the patient's condition more clearly and intuitively to make a clearer diagnosis. However, medical images often suffer from issues, such as low image resolution, similar organizational structure, uneven distribution of foreground and background, which pose great challenges in clinical diagnostics. The traditional segmentation process relies heavily on the experience and energy of physicians, and inevitably leads to misdiagnoses or missed diagnoses. Therefore, quick and efficient image analysis has become a valuable research topic in the field of medical-clinical diagnosis to overcome these significant challenges. The application of artificial intelligence in medical field is multifaceted. Chakraborty et al. [1] proposed a IoMT-based cloud-fog diagnostics for heart disease. Kishor et al. [2] proposes a hybrid Machine Learning Classification Techniques to analyze the complex biomedical data. Chakraborty et al. [3] proposed a reinforcement learning-based method with Medical Information system to improve the quality of service over a heterogeneous network.

Convolutional neural network (CNNs) perform excellently on the medical segmentation task with strong feature extraction and adaptive learning ability. In particular, segmentation models based on Full Convolutional Neural Networks (FCNs) [4] can significantly improve the general technical level of medical image segmentation. However, due to the loss of detailed information in the high-level semantic expression, the up-sampling process of FCNs lacks sufficient spatial detail information, leading to blurred boundaries of the segmentation results. The U-Net [5] integrates semantic information with spatial information using skip connection, which has become the most commonly used structure in medical image segmentation tasks. The reason for the success of U-Net is that the skip connection directly provides an upsampling process with low-level texture information, which is critical for high-level semantic features. Consequently, these connections can provide deconvolution [6] layers with essential high resolution features. Several researchers have made considerable efforts to capture distinguishing features based on U-Net. For example, the U-Net++ [7] can obtain discriminative features through its nesting architecture and dense skip connection. Attention U-Net [8] and Channel U-Net [9], attempt to combine the U-Net with various attention mechanisms with the purpose of enhancing enhancing discriminative features, which have been applied to optimize the significant information extracted from encoders and decoders, respectively. The U-Net [5] ignores the semantic gap in feature fusion, while AUNet [10] proposes an attention-guided upsampling module to optimize the skip connection process, which could remove redundant spatial information and reduce the semantic gap. Due to the variable size and shape of the lesion areas in medical images, uneven distribution of the foreground and background areas, blurred boundaries, and semantic gap, it is still difficult to extract the discriminative features of lesions and improve the effect of feature fusion when decoding. The model proposed in this paper enhances the expressiveness of lesion area features by means of enhancing the expressiveness of spatial features and improving the accuracy of semantic location information. At the same time, semantic features are enhanced at both channel and spatial levels to provide accurate semantic location expression for medical image segmentation, and jointly improve the discrimination of lesion area features.

Recently, the global context modeling capability of Transformer [11], TransUnet [12], and TransAttUnet [13] was found to improve global semantic information on the location and shape of lesions, and thus enhance discriminative semantic feature. DS-TransUnet [14] directly uses the swin Transformer [15] to complete information fusion and disregards the semantic gap between spatial and semantic information. These methods ignore limitations in capturing fine-grained details of the Transformer, especially for medical images, which can still be optimized for discriminative feature extraction and fusion. From the perspective of improving task and semantic position correlations of spatial detail information, our method uses transformer-based multihead attention mechanism to remove redundant information in spatial features and reduce semantic deviation. This in turn optimizes the fusion of spatial and semantic information of the lesion area and improves the accuracy of medical image segmentation.

In this paper, we propose a novel U-Net variant with a transformer called EG-TransUNet, which can effectively preserve reliable discriminative features and achieve effective fusion of spatial and semantic information in U-Net. This is achieved by jointly utilizing the progressive enhancement module (PEM), the semantic guidance attention (SGA), and the channel spatial attention (CSA).

To solve the issue of distinguishing the extraction of features in lesion areas, PEM based on a self-attention mechanism cascades the feedback of various receptive field features of medical images and uses the global self-attention mechanism to obtain more extensive guidance information. CSA captures the global relationship of the self-attention mechanism to calculate the similarity between each channel feature and each spatial position. This in turn enhances semantic position information at the channel and spatial level to obtain more accurate information.

Considering the difficulties in feature fusion of medical image segmentation tasks, SGA starts its process by improving the task and semantic position correlation of detailed spatial information and fully exploits prior knowledge of medical images in the skip connection part. The relationship between semantic and spatial details is fully explored and improved, the redundant information is removed, and the subsequent feature fusion process is optimized.

We evaluated the effectiveness of the proposed EG-TransUNet using four typical medical image segmentation tasks covering polyp segmentation [13, 14], nuclei segmentation [16], melanoma segmentation [17], and gland segmentation (GLAS) [18], and the experimental results demonstrated the consistent effectiveness of the proposed EG-TransUNet. Our main contributions are summarized as follows:

1. This paper proposes a Transformer-based U-shaped framework called EG-TransUNet, which exploits three novel modules, namely the PEM, SGA, and channel spatial attention (CSA), to improve the performance of medical segmentation.
2. PEM consists of dilated self-attention convolution (DSA) and gated convolution (GC), which can capture spatial features of the target region. CSA can capture long-range contextual information in channel and spatial dimensions using self-attention. SGA is used to remove redundant information and reduce the semantic gap.
3. In comparison to certain state-of-the-art methods, the effectiveness and generalizability of the proposed EG-TransUnet is demonstrated by extensive experiments on medical image segmentation that consistently showed that the proposed method outperforms these previous methods, especially when it comes to polyp segmentation tasks.

The remaining of this paper is organized as follows: “Related work” Section provides an overview of some related works on automatic medical image segmentation, and “Methods” Section describes the proposed EG-TransUNet in detail. Next, comprehensive experiments and ablation studies are presented in “Experimental analysis” and “Generalization and discussion” sections. Finally, “Conclusion” section summarizes the present work.

## Related work

In this section, we provide a brief overview of research related to medical image segmentation tasks. We first summarize the most typical U-shaped CNNs methods in medical image segmentation and then review the application of vision transformers in recent years, especially in image segmentation tasks.

### Medical image segmentation based on CNNs

CNNs, particularly U-Net [5] and its encoder-decoder-based variants, have proven their exceptional performance in segmenting medical images.

In order to successfully detect and segment each individual breast slice in the DCE-MRI breast tumor dataset, Benjelloun et al. [19] developed a fully convolutional neural network architecture based on U-Net [5] for the first time. Consequently, some studies attempted to combine the low-level feature of the shallow layer with the high-level feature of the deep layer to take full advantage of multiscale information and ameliorate detail restoration issues. U-Net++ [7], U-Net 3+ [20] and DenseUNet [21] used full-scale skip connections and deep supervisions to learn hierarchical representations from full-scale aggregated feature maps. U2-Net [22] was able to capture more contextual information from different scales with a mixture of different receptive field sizes in the proposed Residual U Blocks (RSU). KiU-Net [23] introduced a novel structure that could project data to higher dimensions and obtain both incomplete and complete features that improved segmentation of small anatomical structures. Furthermore, MA-UNet [24] established a multiscale mechanism to remove semantic ambiguity in skip connections by adding attentional gates (AGs) which can explicitly model the relationship between channels. In addition, MA-U-Net used multiscale predictive fusion to exploit global information at different scales by combining local features with their corresponding global dependencies. Finally, DoubleU-Net [25] used two U-Net and an atrous spatial pyramid pooling [26] to obtain accurate spatial, semantic, and contextual features.

Thereafter, many attention-guided methods have been proposed to optimize the segmentation performance of U-Net by enhancing the discriminative features in medical images obtained from different imaging modalities. Oktay et al. [8] proposed a novel AG mechanism based on U-Net that allowed the model to focus on targets of different shapes and sizes. Chen et al. [9] proposed a spatial channel-wise convolution, which a convolution along the direction of the channel of feature maps, to extract the relationship of spatial information between pixels, and thus discriminate the lesion areas. Tang et al. [27] proposed a criss-cross attention module to capture rich global context information in both horizontal and vertical directions for all pixels, thus facilitating accurate lung segmentation. Chen et al. [28] used the Aggregated Residual Transformations to learn a robust and expressive feature representation. The soft attention mechanism was then applied to improve the capability of the model to discriminate a variety of symptoms of the COVID-19 in chest CT. Tomar et al. [29] proposed a feedback attention network (FANet) that unified the previous epoch mask with the feature map of the current training epoch, allowing the predictions to be iteratively corrected during testing time.

Altogether, current studies often improve the expressiveness of feature and optimize the use of spatial information in skip connections through multiscale feature fusion and attention mechanisms. The U-Net++ model does not consider semantic bias when fusing features at different scales, but instead obtains the optimal solution using a simple dense connection search, and fails to optimize the process of semantic feature extraction. Therefore, the extracted features of the lesion area still suffer from insufficient discrimination. However, multiscale feature fusion may cause valuable detail loss and may suffer from information redundancy. Attention mechanisms often fail to recognize the boundaries of images with similar organizational structures, leading to the loss of available feature representation.

### Transformers in medical segmentation

Transformers [11] have triggered great achievements in the field of computer vision due to their ability to model long-range contextual interactions. In medical image segmentation, TransUNet [12] proved that Transformers could serve as powerful encoders for medical image segmentation tasks, with the combination of U-Net to enhance finer details by recovering localized spatial information. TransFuse [30] combined Transformers and CNNs in a parallel style, where both global dependency and low-level spatial details could be efficiently captured and fused in a much shallower manner. MedT [31] proposed a gated axial attention model that used a transformer-based gating position-sensitive axial attention mechanism to segment medical images based on Axial-DeepLab [32]. In TransAttUnet [13], multilevel guided attention and multiscale skip connection were co-developed to effectively improve the functionality and flexibility of the traditional U-shaped architecture. DS-TransUNet [14] applied the swin-transformer block [15] to both the encoder and the decoder. This was probably the first attempt to simultaneously incorporate the advantages of hierarchical Swin Transformer into both the encoder and the decoder of the standard U-shaped architecture with the purpose of enhancing the segmentation quality of varying medical images.

However, the above medical image segmentation model fails to take full advantage of the spatial detail information of the lesion area, resulting in low accuracy in medical image segmentation tasks in complex environments. Although the TransUnet and TransAttUnet models use the transformer structure to enhance the global expression of features, they only focus on the acquisition of semantic location information and do not improve the acquisition process of spatial features. Therefore, these models cannot use distinctive feature texture during decoding. On one hand, the DS-TransUNet model uses the swin transformer model to complete information fusion, which completely ignores the semantic deviation between spatial and semantic information. On the other hand, the swin transformer structure lacks interpretability in the segmentation process.

Inspired by these approaches, we propose a U-shaped structure called EG-TransUNet that applies a Transformer, specifically multihead attention, to both the encoder and the decoder. We believe that this Transformer-based structure can outperform previous models and optimize medical image segmentation.

## Methods

This section explicitly introduces the proposed EG-TransUNet. First, an overview of the proposed EG-TransUNet is presented. Then, we present the principles and structure of EG-TransUNet, followed by a detailed description of each component. Finally, we elaborate the loss function used in our EG-TransUNet.

### Overview of the EG-TransUNet

**Fig. 1:**
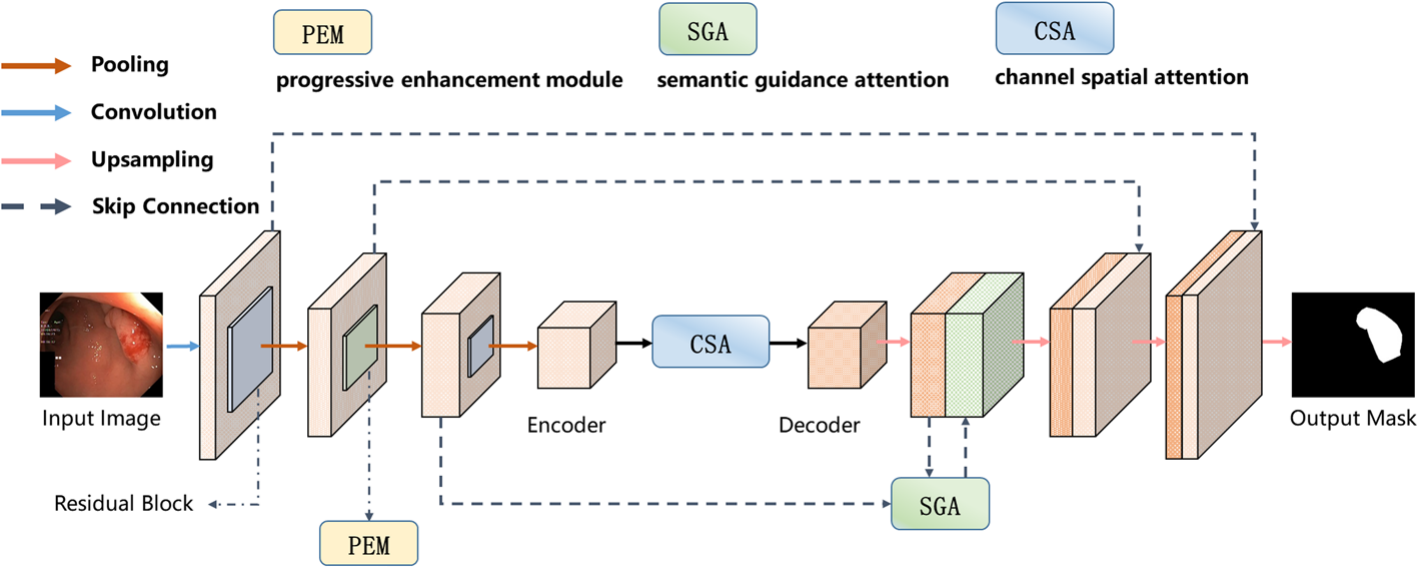
Illustration of the proposed EG-TransUNet for medical image segmentation

The input of medical image is $X \in {\mathbb{R}}^{C \times H \times W}$, where $C$ is the number of channels and $H \times W$ represents the spatial resolution of image. Consequently, the goal of medical image segmentation task is to predict the corresponding pixel-wise semantic label maps with $H \times W$ size. Consistent with the previous work on medical image segmentation tasks, the EG-TransUNet is also built on a U-shaped architecture, whose brief structure is illustrated in Fig. 1.

**Fig. 2:**
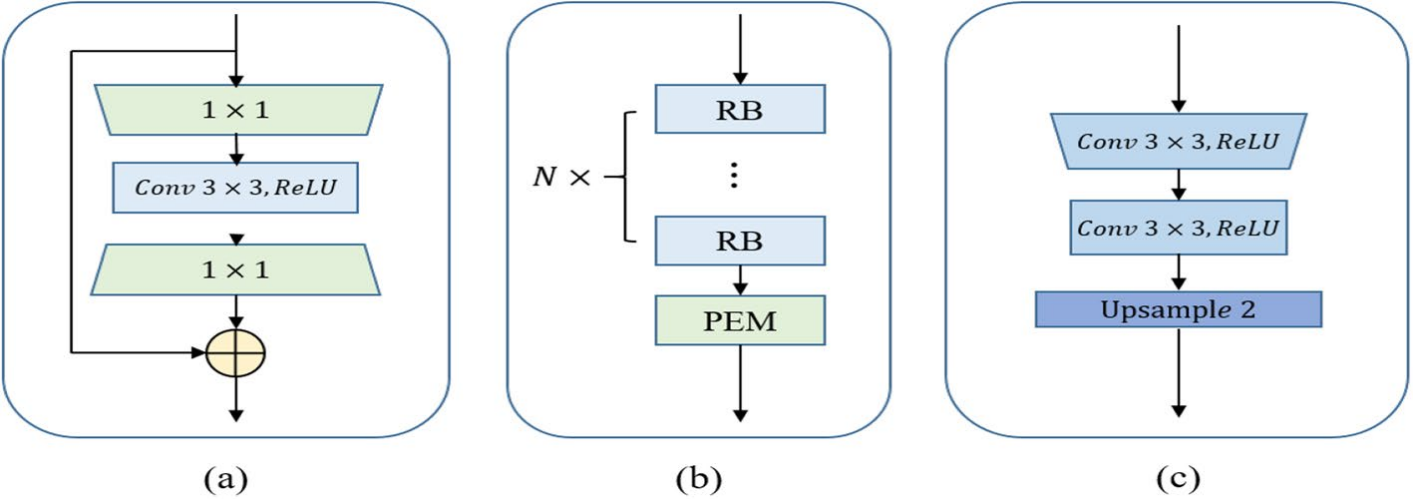
Illustration of the encoding and decoding. **a** The residual block (RB) used in the process of encoding. **b** The structure of each encoding and the location of PEM. **c** The structure of each decoding

The EG-TransUNet consists of three processes, namely encoding, decoding and semantic feature enhancement. The encoding process uses ResNet50 [33] as the backbone to obtain feature from different receptive fields of the input medical images. The structure of the residual block is shown in Fig. 2a. The combination of the latter two blocks of the ResNet50 [33] network generates four encoding blocks, each of which downsamples the feature maps by a factor of two. The structure of each encoding process and the location of PEM are shown in Fig. 2b. The decoding process is consistent with the standard U-Net and constructs the segmentation results on a step by step approach, including two convolutions and one upsampling, as shown in Fig. 2c. The semantic feature enhancement is used to improve the representation ability of the semantic feature, which refers to the CSA in Fig. 1. Considering the computation cost, we only embed PEM and SGA into the third encoding and the second decoding processes, respectively.

### Progressive enhancement module (PEM)

**Fig. 3:**
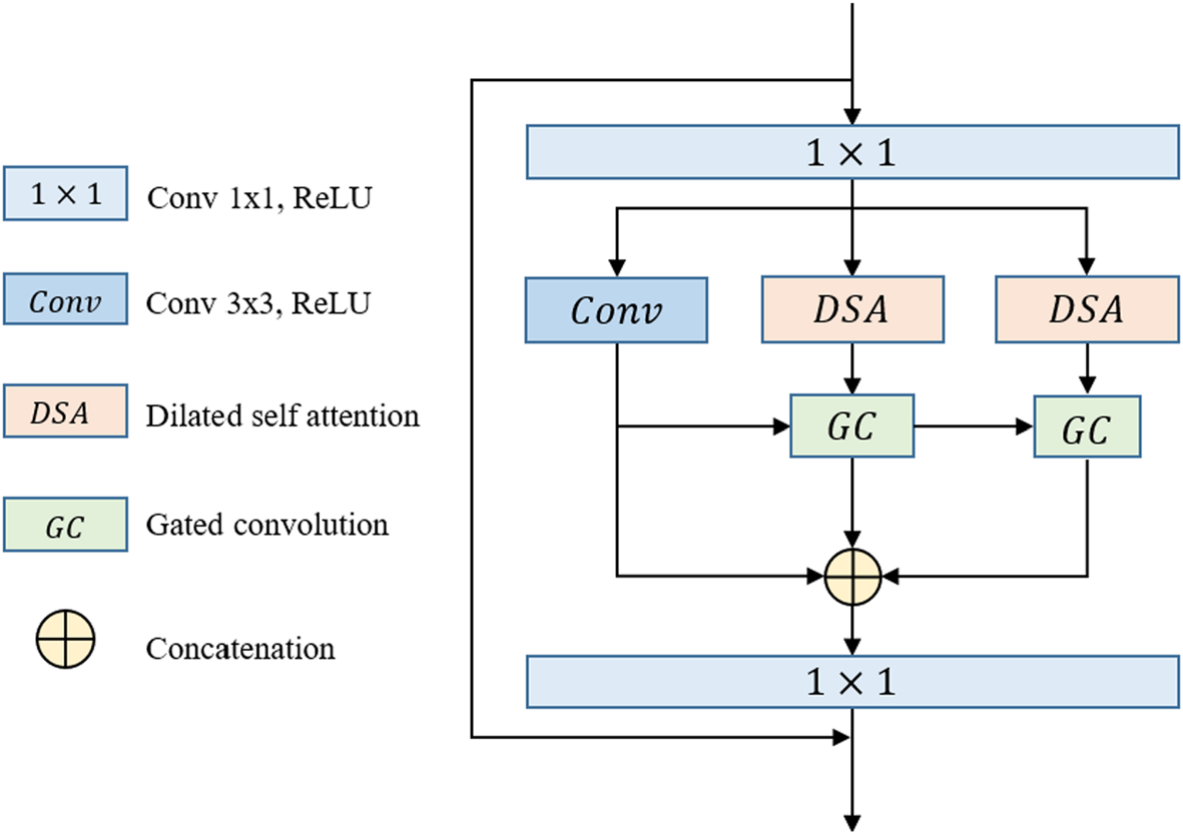
Illustration of the proposed progressive enhancement module (PEM)

PEM consists mainly of two parts, namely dilated self-attention convolution and gated convolution. As shown in Fig. 3, we use one 3 × 3 convolution operation and two dilated self-attention convolution modules with dilated 3 × 3 convolution rates of two and three, respectively, to obtain features from different receptive fields. Then, we feed the features obtained by the 3 × 3 convolution operation and the dilated self-attention convolution module with a dilated rate of two into the GC, and make the larger receptive field feature guide the discriminative extraction process of the original feature. Consequently, the feature from the first GC module and the feature from the dilated self-attention convolution module with a dilated rate of three are fed into the GC again, and the discriminative feature is further extracted. Finally, the original 3 × 3 convolution feature is combined with the output feature of the two-GCs as the final output.

1. *Dilated Self-attention Convolution*: The DSA is built on the multihead self-attention of a Transformer and allows the model to care only for information stemming from global representation subspaces. We use convolution embedding instead of linear embedding, so that the DSA cannot only aggregate global contextual information, but also account for local spatial information. Compared to traditional convolution, dilated convolution can flexibly change the receptive field by changing the rate of dilation while ensuring the consistency of the feature size. The DSA can selectively aggregate the global context to the learned feature and encode broader contextual positional information into the local feature using convolutional embedding and matrix multiplication, which can improve intraclass compactness and optimize feature representations.

**Fig. 4:**
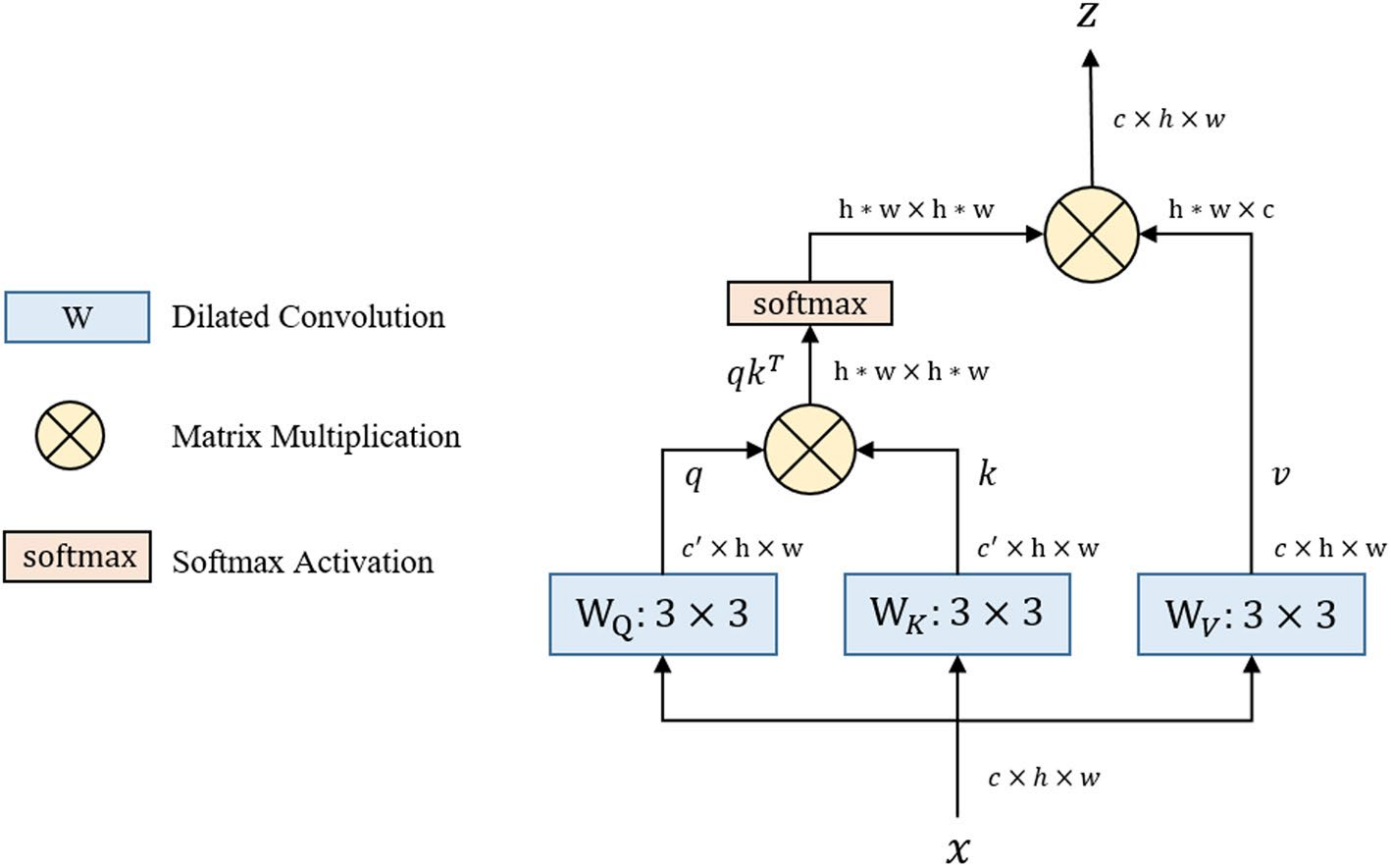
Illustration of the proposed dilated self-attention convolution (DSA)

The pipeline of the dilated DSA component is depicted in Fig. 4, and we refer to the description of the TransAttUnet Transformer to describe DSA.

First, we apply three dilated convolution operations on the encoder feature $x$ to generate the feature maps q, k, and v. Subsequently, we reshape $q$ and $k$ and perform a matrix multiplication with softmax normalization, resulting in the position relevance attention map. T he above operation can be defined as follows:

1 $$\begin{aligned} M & = reshape\left( q \right) \\ N & = reshape\left( k \right) \\ T & = reshape\left( v \right) \\ B_{i,j} & = \frac{{\exp \left( {M_{i} \cdot N_{j} } \right)}}{{\sum\nolimits_{k = 1}^{n} {\exp \left( {M_{k} \cdot N_{j} )} \right)} }} \\ DSA\left( {B,T} \right) & = B \cdot T \\ \end{aligned}$$

where $B_{i,j}$ measures the impact of the $i_{th}$ position on the $j_{th}$ position, $n = h \times w$ is the number of pixels, and $M$, $N$, and $T$ represent the reshaped features. $B$ represents the position relevance attention map. Then, $T$ is multiplied by $B$, and we reshape the optimized feature maps to obtain the output of DSA.

(2) *GC*: The gated convolution module consists of two inputs, indicating one large and one small receptive field feature, as shown in Fig. 3. Then, two different convolutional operations are applied to the input features to generate the gate maps. Finally, a multiplication operation is performed to obtain the final output. The calculation process can be formulated as follows:
  2 $$\begin{gathered} Gate = W_{g} \cdot F_{high} \hfill \\ F = W_{f} \cdot F_{low} \hfill \\ G = \emptyset (F)*\sigma (Gate) \hfill \\ \end{gathered}$$
  where $W_{g}$ and $W_{f}$ are the embedding matrices of different convolution projections and $F_{high}$ and $F_{low}$ represent two inputs. $Gate$ is the attention map and $\sigma$ is the $sigmoid$ function, which maps all values to the interval between 0 and 1. Finally, $F$ is the feature embedding and $\emptyset$ means $ReLU$ activation.

### Channel spatial attention

**Fig. 5:**
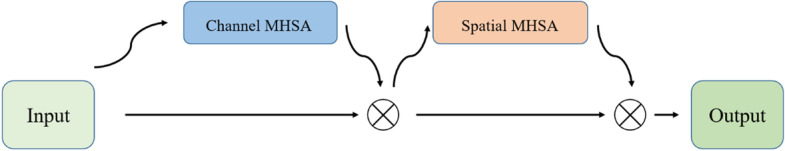
Illustration of the proposed channel spatial attention (CSA)

The CSA helps our model to capture the wider and richer contextual representations and obtain more accurate semantic location representation of the lesion region. Inspired by CBAM [34], the two self-attention mechanisms, i.e., Channel and Spatial MHSA (multihead self-attention) are connected in series to form the CSA module, as shown in Fig. 5.

Both channels can use the self-attention mechanism to calculate the global correlation between channel feature and spatial features and enhance channel and spatial information under the guidance of autocorrelation as used in transformer [11]. Specifically, we use absolute position embedding to capture the spatial relationship of features in spatial MHSA, while no position embedding is used in channel MHSA. The process of absolute position embedding [35] can be formulated as follows:

3 $$\begin{aligned} e_{i,j} & = \frac{{\left( {x_{i} W^{q} } \right) \cdot \left( {x_{j} W^{k} } \right)^{T} }}{{\sqrt {d_{z} } }} \\ p_{i,j} & = \left( {x_{i} W^{v} } \right) \cdot \left( {a_{i,j}^{k} } \right)^{T} \\ \alpha_{i,j} & = \frac{{\exp \left( {e_{i,j} + p_{i,j} } \right)}}{{\sum\nolimits_{k = 1}^{n} {\exp \left( {e_{i,k} + p_{i,k} } \right)} }} \\ z_{i} & = \sum\nolimits_{j = 1}^{n} {\alpha_{i,j} \cdot x_{j} } \\ \end{aligned}$$

Each output element, $z_{i}$, is computed as a weighted sum of a linearly transformed input elements. $W^{q}$, $W^{k}$, and $W^{v}$ are parameter matrixes, which are unique for each layer and attention head. Each weight coefficient $\alpha_{i,j}$ is computed using a softmax function, while $e_{i,j}$ represents the correlation between two input elements, which is computed by the scaled dot product. The absolute position embedding between the input elements $x_{i}$ and $x_{j}$ is reflected by the matrix $p_{i,j}$, which is shared across the attention heads and optimized by backward propagating. Finally,$a_{i,j}^{k}$ is a trainable position parameter matrix.

### Semantic guide attention (SGA)

**Fig. 6:**
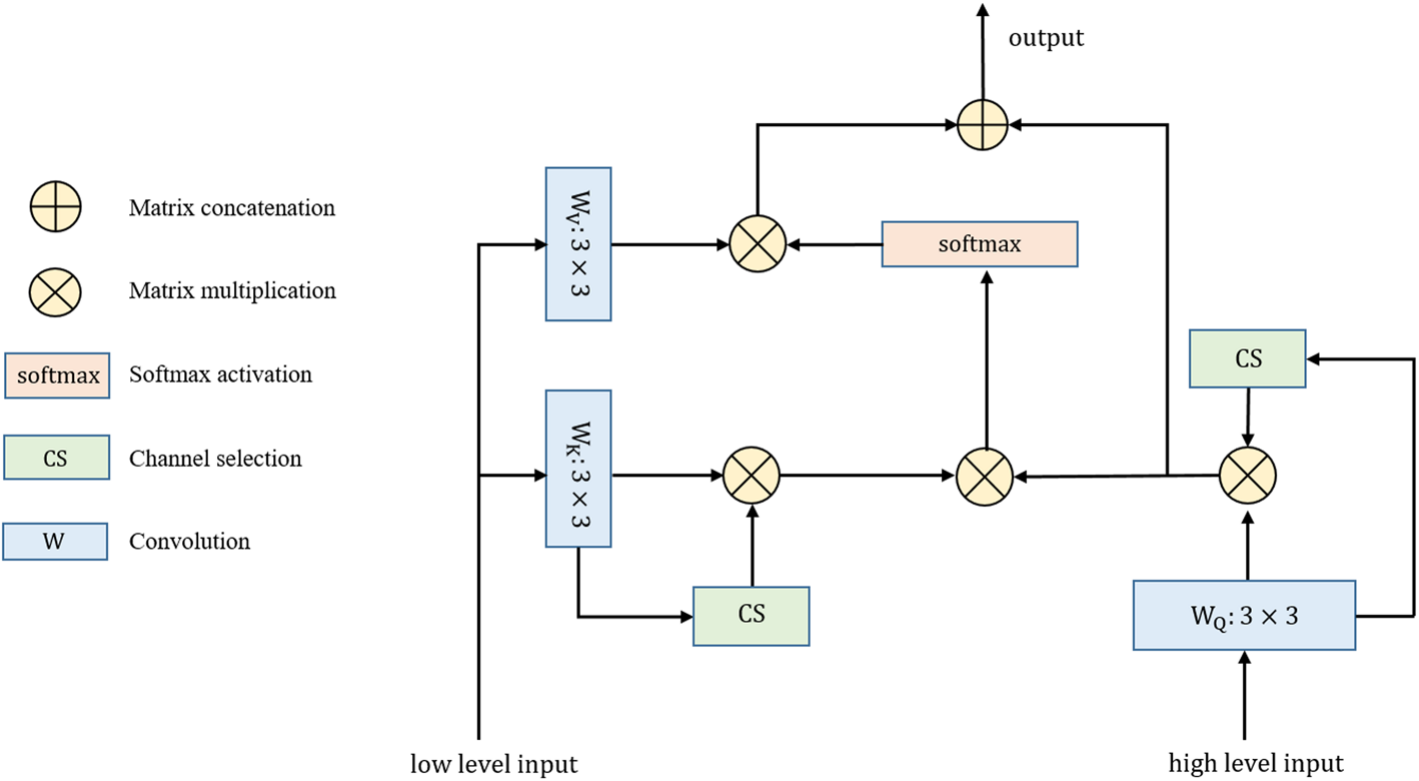
Illustration of the proposed semantic guidance attention (SGA)

Pertaining to the decoding process, the MHSA can calculate the correlation between the corresponding positions between spatial and semantic features. Hence, the SGA can remove redundant textual information, reduce the semantic gap, and improve the effect of feature fusion in skip connection with the correlation. The pipeline of SGA is depicted in Fig. 6, demonstrating that the overall structure is similar to the self-attention mechanism.

The low-level input from the encoder feature is firstly reshaped to generate K and V, respectively. Then, channel selection (CS) is applied to obtain the important channels of K. The high-level input from the decoder feature is reshaped into the Q matrix and CS is applied to select the important channels. A multihead scaled dot-product operation with softmax normalization between Q and the transposed version of K is conducted to generate the contextual attention map, which represents the global similarities of the given elements from the semantic and spatial features. To obtain the aggregation of values weighted by contextual attention, the map should be multiplied by V. Finally, we concatenate the reshaped low-level and high-level features to obtain the final output of SGA.

The channel selection (CS) can be formulated as follows:

4 $$\begin{aligned} P_{c} & = \frac{1}{h \times w}\sum\limits_{i = 1}^{h} {\sum\limits_{j = 1}^{w} {(F_{c} (i,j))} } ,P \in {\mathbb{R}}^{c} ,F \in {\mathbb{R}}^{c \times h \times w} \\ A_{w} & = sigmoid(W \cdot P),W \in {\mathbb{R}}^{c \times c} ,A_{w} \in {\mathbb{R}}^{c} \\ \widetilde{F} & = A_{w} \cdot F,F \in {\mathbb{R}}^{c \times h \times w} ,\widetilde{F} \in c \times h \times w \\ \end{aligned}$$

where $F_{c}$ is the feature of the $C_{th}$ channel of feature $F$. $W$ refers to the weight, which is constantly optimized in the model training process, enabling the key channel feature to be accurately selected. $A_{w}$ refers to the task correlation of all channel features. Finally, the $A_{w}$ is multiplied by the input feature $F$ to obtain the key channel feature $\widetilde{F}$.

### Loss function

During the training phase, the EG-TransUNet uses an end-to-end training manner. We have used binary cross-entropy loss $L_{BCE}$ and dice loss $L_{Dice}$. The calculation formulas of $L_{BCE}$ and $L_{Dice}$ are as follows:

5 $$\begin{aligned} L_{BCE} & = - \sum\limits_{i = 1}^{n} {\left( {y_{i} \log (p_{i} ) + (1 - y_{i} )\log (1 - p_{i} )} \right)} \\ L_{Dice} & = 1 - \frac{{\sum\nolimits_{i = 1}^{n} {y_{i} p_{i} + \varepsilon } }}{{\sum\nolimits_{i = 1}^{n} {\left( {y_{i} + p_{i} } \right) + \varepsilon } }} \\ L_{Total} & = \alpha \cdot L_{BCE} + \beta \cdot L_{Dice} \\ \end{aligned}$$

where n is the total number of pixels in each image, $y_{i}$ represents the ground-truth value of the $i_{th}$ pixel, and $p_{i}$ represents the confidence score of the $i_{th}$ pixel in the prediction results. In our experiment, $\alpha = \beta = 0.5$, and $\varepsilon = 10^{ - 6}$.

## Experimental analysis

In this section, we introduced five segmentation datasets and conducted some experiments to compare our proposed model with SOTA methods.

### Description of data sets

**Fig. 7:**
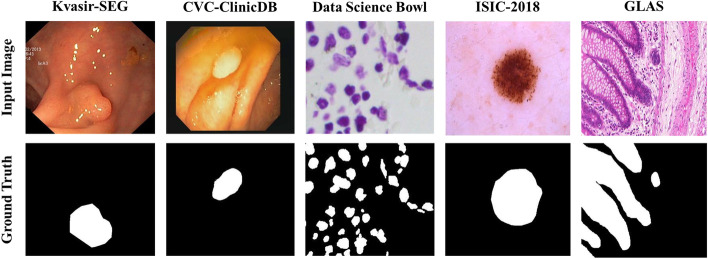
Illustration of examples of medical images with the corresponding semantic segmentation annotations

To evaluate the effectiveness of EG-TransUNet, we used five public biomedical datasets namely Kvasir-SEG [36], CVC-ClinicDB [37], 2018 Data Science Bowl [16], ISIC-2018 Challenge [17] and 2015 MICCAI Gland Segmentation (GLAS) [18]. An example of each dataset can be found in Fig. 7.

1. Kvasir-SEG [36]: The Kvasir-SEG is a dataset of gastric polyp images for developing applications on the automated diagnosis of polyps from endoscopic images. It is an extension of Kvasir [38] which contains images from the inside of the gastrointestinal (GI) tract. The Kvasir-SEG contains 1000 images with the corresponding annotations and these images are randomly split into 800 images for training, 100 images for validation and 100 images for testing.
2. CVC-ClinicDB [37]: The CVC-ClinicDB is a dataset of 612 images from 31 colonoscopy sequences with a resolution of 384 × 288. It is used for polyp segmentation in colonoscopy videos. These images are randomly split into 490 images for training, 61 images for validation and 61 images for testing.
3. 2018 Data Science Bowl [16]: The purpose of this dataset was to find the nuclei in divergent images, including a total of 670 images. These images are randomly divided into 536 images for training, 67 images for validation, and 67 images for testing.
4. ISIC -2018 Challenge [17]: The ISIC-2018 is a comprehensive dataset of dermoscopy images for developing applications on the automated diagnosis of melanoma using dermoscopic images. Their work [17] focuses on lesion segmentation from dermoscopic images acquired with a variety of dermatoscopy types. It contains 2596 images with the corresponding annotations, and these images are randomly split into 2078 images for training, 259 images for validation, and 259 images for testing.
5. GLAS [18]: The GLAS dataset is published by the Colon Histology Images Challenge Contest of MICCAl’2015 and consists of 165 colon histology images derived from 16 H&E stained histological sections of stage T3 or T4 colorectal adenocarcinomas from different patients. In particular, each sample is processed on different occasions in the laboratory, resulting in high inter-subject variability in both stain distribution and tissue architecture. In our experiments, the GLAS dataset is split into two subsets: 85 images for training and 80 for testing, which is consistent with previous works [23, 31]

### Evaluation metrics

To compare our proposed EG-TransUNet to the SOTA methods, the standard evaluation indicators used included the Dice coefficient (Dice) [39], intersection over Union (IoU), precision and recall, which are related to four values, namely true positive (TP) true negative (TN) false positive (FP) and false negative (FN), respectively.

6 $$\begin{aligned} Dice & = \frac{2 \times TP}{{2 \times TP + FP + FN}} \\ IoU & = \frac{2 \times TP}{{TP + FP + FN}} \\ \Pr ecision & = \frac{TP}{{TP + FP}} \\ {\text{Re}} call & = \frac{TP}{{TP + FN}} \\ \end{aligned}$$

### Implementation details

We implemented the proposed EG-TransUNet using PyTorch [40], and all experiments were conducted on a NVIDIA GeForce 3090 GPU with 12 GB memory. Furthermore, we adopted the stochastic gradient descent optimizer with a momentum of 0.9 and a weight decay 0.001 to optimize the training process.

For each dataset, the images were resized into 320 × 320. Data augmentation, such as random cropping, random rotation, horizontal flipping, vertical flipping, and grid distortion, were also used. Furthermore, the EG-TransUNet was trained for 300 epochs with a batch size of four. Besides, the initial learning rate was $5e^{ - 3}$, decaying by a factor of 10 for every 40 epochs.

### Results

1. Comparison on Kvasir-SEG

**Fig. 8:**
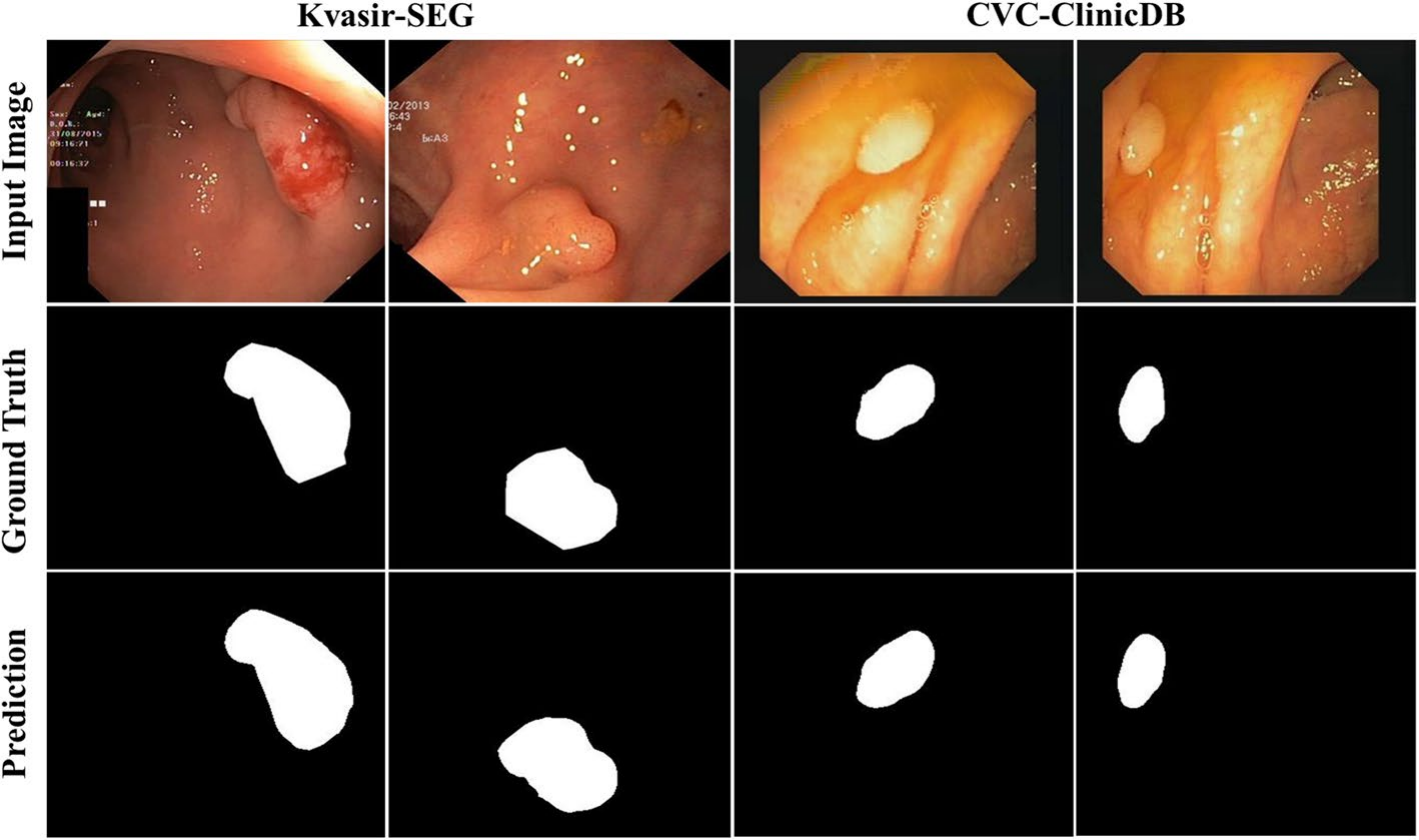
Qualitative results of EG-TransUNet for Kvasir-SEG and CVC-ClinicDB

In our experiment, we selected two popular colonoscopy datasets of which the first one was Kvasir-SEG. Compared to other models, our quantitative results on the Kvasir-SEG dataset achieved SOTA performance, as presented in Table 1 and Fig. 10. Compared to MSRF-Net [41], our results demonstrated that Kvasir-SEG could be improved by 1.27% on mDice and 0.13% on mIoU, respectively. Our method also achieveed an improvement of 0.41% on the recall compared to DS-TransUNet-L [14]. Although MSRF-Net [41] was slightly ahead with respect to precision, our method provided significantly better results in the other three indices, suggesting that this model could achieve a more balanced and excellent segmentation effect. The perfect qualitative result compared to the ground truth can be observed in Fig. 8.

(2) Comparison on CVC-ClinicDB

**Table 1:** Comparisons with the state-of-the-art baselines on the Kvasir-SEG dataset terms

Method	Year	mDice	mIoU	Recall	Precision
ResUNet [42]	2018	0.7907	0.4287	0.6909	0.8713
ResUNet++ [43]	2019	0.8133	0.7927	0.8774	0.7064
U-Net [5]	2015	0.8180	0.7460	0.6306	0.9222
U-Net++ [7]	2018	0.8210	0.7430	–	–
HRNetV2-W48 [44]	2020	0.8896	0.8262	0.8973	0.9056
DS-TransUNet-B [14]	2021	0.9110	0.8561	0.9352	0.9143
DS-TransUNet-L [14]	2021	0.9130	0.8592	0.9360	0.9164
TransFuse [30]	2021	0.9180	0.8680	–	–
MSRF-Net [41]	2021	0.9217	0.8914	0.9198	**0.9666**
EG-TransUNet (ours)	**–**	**0.9344**	**0.8927**	**0.9401**	0.9436

The “–” denotes the corresponding result is not provided. For each column, the best results are highlighted

The second colonoscopy dataset was CVC-ClinicDB on which we achieved SOTA performance compared to other models, as shown in Table 2. Our model achieved an mDice of 0.9523, which corresponded to a 1.01% improvement in mDice over the best performing DS-TransUNet-L [14]. We also found that mIoU was 0.9130, reflecting an improvement of 0.87% over the SOTA performance of MSRF-Net [41]. In addition, EG-TransUNet achieved precision and recall values of 0.9536 and 0.9540, respectively, values that are competitive with the best performing MSRF-Net and DoubleU-net [25]. Figure 8 demonstrates that our method produced almost exactly the same boundaries and shapes as the ground truth masks. The two above experiments revealed that our method could identify the lesion area in colonoscopy image data more accurately compared to conventional models, which offer a small segmentation target and blurred boundaries.

(3) Comparison on 2018 Data Science Bowl

**Table 2:** Comparisons with the state-of-the-art baselines on the CVC-ClinicDB dataset

Method	Year	mDice	mIoU	Recall	Precision
FCN [45]	2017			0.7732	0.8999
CNN [46]	2018	0.87	–	–	–
SegNet [47]	2018	–	–	0.8824	–
U-Net [5]	2015	0.8781	0.7881	0.7865	0.9329
ResUNet++ [43]	2019	0.9199	0.8892	0.9391	0.8445
DoubleU-Net [25]	2020	0.9239	0.8611	0.8457	**0.9592**
TransUNet [12]	2021	0.9350	0.8870	–	**–**
DS-TransUNet-B [14]	2021	0.9350	0.8845	0.9464	0.9306
DS-TransUNet-L [14]	2021	0.9422	0.8939	0.9500	0.9369
MSRF-Net [41]	2021	0.9420	0.9043	**0.9567**	0.9427
EG-TransUNet	**–**	**0.9523**	**0.9130**	0.9540	0.9536

The “–” denotes the corresponding result is not provided. For each column, the best results are highlighted

**Fig. 9:**
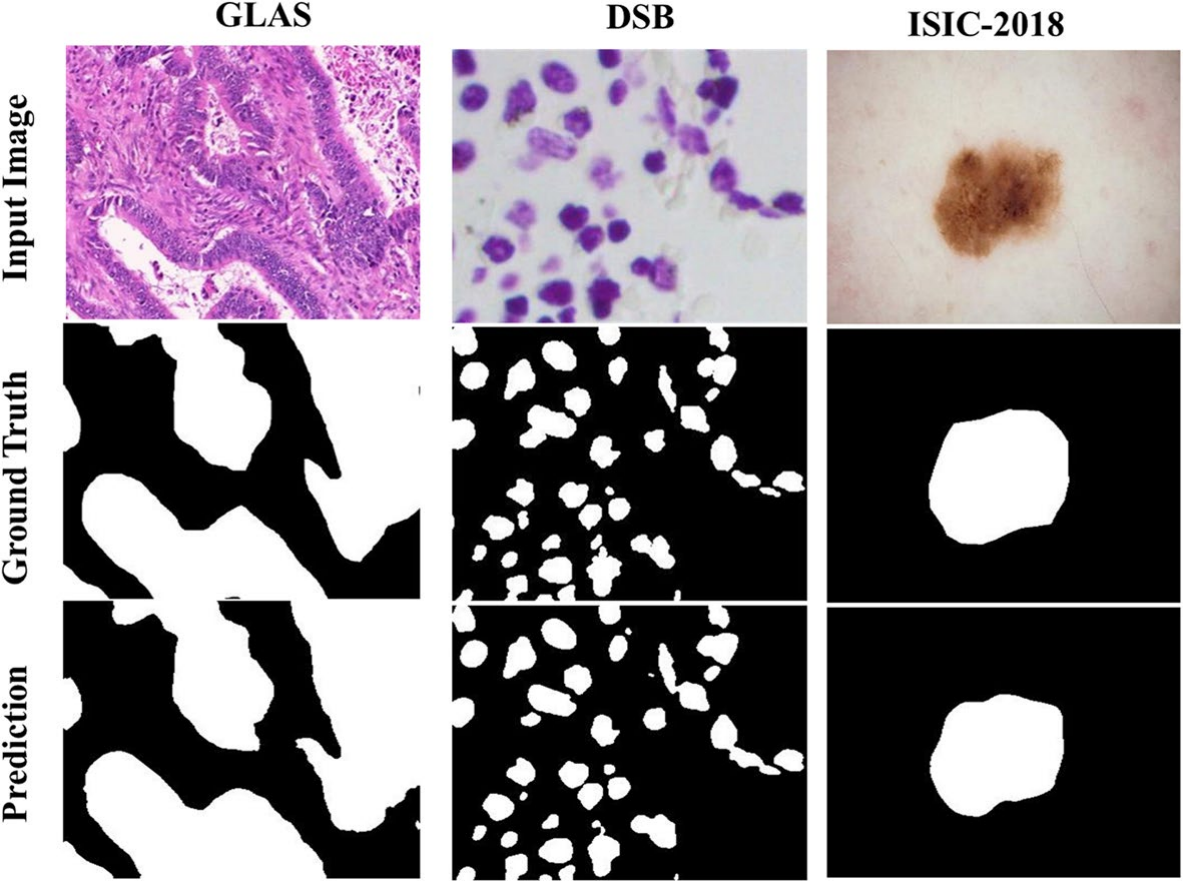
Qualitative results of EG-TransUNet for GLAS, DSB and ISIC-2018

Table 3 shows the comparison results of the proposed EG-TransUNet with some of the presented approaches on the 2018 Data Science Bowl dataset. We obtained an mDice value of 0.9349, mIoU of 0.8908, recall of 0.9482, and precision of 0.9336, which outperformed the best performing DoubleU-Net, MSRF-Net, and DS-TransUNet in most metrics. The qualitative results shown in Fig. 9 show that our predictions were almost identical to the ground truth masks. Our model is also suitable for data sets with a large number of irregularly distributed targets and blurred boundaries, maintaining high performance and providing accurate results for clinical medical image analysis.

(4) Comparison on ISIC-2018 Skin Lesion Segmentation challenge

**Table 3:** Comparisons with the state-of-the-art baselines on the 2018 data science bowl (DSB) dataset

Method	Year	mDice	mIoU	Recall	Precision
U-Net [5]	2015	0.7573	0.9103	–	–
PraNet [48]	2020	0.8103	0.7108	0.8062	0.8231
U-Net++ [7]	2018	0.8974	**0.9255**	–	–
DoubleU-Net [25]	2020	0.9133	0.8407	0.6407	0.9406
TransAttUnet_R [13]	2021	0.9162	0.8498	0.9185	0.9193
DS-TransUNet-B [14]	2021	0.9200	0.8589	0.9427	0.9054
DS-TransUNet-L [14]	2021	0.9219	0.8612	0.9378	0.9124
MSRF-Net [41]	2021	0.9224	0.8534	0.9402	0.9022
EG-TransUNet	**–**	**0.9349**	**0.8908**	**0.9482**	**0.9336**

The “–” denotes the corresponding result is not provided. For each column, the best results are highlighted

The quantitative comparison results of ISIC-2018 are presented in Table 4, and the corresponding qualitative results are illustrated in Fig. 9. Our method achieved an mDice value of 0.9075, mIoU of 0.8441, and recall of 0.9169, reflecting an improvement of 2.62%, 1.16%, and 2.26%, respectively, over MSRF-Net. Moreover, our model obtained a precision value of 0.9165, which is competitive with other models. Our qualitative results revealed that our model could accurately segment skin lesions of varying sizes and shapes.

(5) Comparison on GLAS

**Table 4:** comparisons with the state-of-the-art baselines on the isic-2018 dataset

Method	Year	mDice	mIoU	Recall	Precision
U-Net [5]	2015	0.6740	0.5490	0.7080	–
PraNet [48]	2020	0.8746	0.8023	0.9128	0.8759
MSRF-Net [41]	2021	0.8813	0.8325	0.8903	0.9267
DoubleU-Net [25]	2020	0.8962	0.8212	0.8780	0.9459
TransAttUnet_D [13]	2021	0.9014	0.8304	0.9042	0.9217
TransAttUnet_R [13]	2021	0.9074	0.8380	0.9093	0.9242
EG-TransUNet	**–**	**0.9075**	**0.8441**	**0.9169**	**0.9165**

The “–” denotes the corresponding result is not provided. For each column, the best results are highlighted

The quantitative comparison results of GLAS are presented in Table 5, and the corresponding qualitative results are illustrated in Fig. 9. Our method achieved an mDice value of 0.9003, mIoU of 0.8247, recall of 0.9025, and precision of 0.9027. Compared to DS-TransUNet, our method improved mDice and mIoU by 2.84% and 4.02%, respectively. The qualitative results obtained suggested that our model could accurately segment glands of varying sizes and shapes.

**Table 5:** Comparisons with the state-of-the-art baselines on the GLAS dataset terms

Method	Year	mDice	mIoU	Recall	Precision
SegNet [47]	2018	0.7861	0.6596	–	–
U-Net [5]	2015	0.7976	0.6763	–	–
ResUNet [42]	2018	0.8088	0.6911	0.8511	0.8001
MedT [31]	2021	0.8102	0.6961	–	–
U-Net++ [7]	2018	0.8113	0.6961	–	–
Attention U-Net [8]	2018	0.8159	0.7006	–	–
KiU-Net [23]	2020	0.8325	0.7278	–	–
DS-TransUNet [14]	2021	0.8719	0.7845	–	–
EG-TransUNet	**–**	**0.9003**	**0.8247**	**0.9025**	**0.9027**

The “–” denotes the corresponding result is not provided. For each column, the best results are highlighted

## Generalization and discussion

In medical imaging, the generalization ability refers to the adaptability of algorithms on datasets from different institutions. In this paper, we used the Kvasir-SEG for training the model, which was then tested on CVC-ClinicDB. Similarly, we conducted this study on an opposite setup as well, i.e., training on CVC-ClinicDB and testing on Kvasir-SEG. Tables 4 and 7 show the results of the generalization study. Furthermore, we discuss ablation studies in detail and present a visual analysis.

### Generalizability results on CVC-ClinicDB

The Table 6 shows the generalization performance results of our model trained on Kvasir-SEG and tested on CVC-ClinicDB. Our EG-TransUNet achieved an mDice value of 0.8939, a mIoU of 0.8420, a recall of 0.9020, and a precision of 0.9147. All the above results demonstrated that our model had higher generalizability than other SOTA methods. Moreover, the high recall value obtained indicates that our model has high medical sensitivity and can effectively reduce the rates of missed diagnoses.

**Table 6:** Generalizability results of the models trained on Kvasir-SEG and tested on CVC-Clinicdb

Method	Year	mDice	mIoU	Recall	Precision
U-Net [5]	2015	0.6302	0.5015	0.5612	0.8249
U-Net++ [7]	2018	0.4267	0.3623	0.4337	0.6877
HRNetV2-W18-Smallv2 [44]	2020	0.6428	0.5513	0.6811	0.7253
HRNetV2-W48 [44]	2020	0.7901	0.6953	0.8796	0.7694
MSRF-Net [41]	2021	0.7921	0.6498	0.9001	0.7694
EG-TransUNet	**–**	**0.8939**	**0.8420**	**0.9020**	**0.9147**

### Generalizability results on Kvasir-SEG

The corresponding generalization performance results of our model trained on the CVC-ClinicDB dataset and tested on the Kvasir-SEG dataset are shown in Table 7. Our EG-TransUNet obtained an mDice value of 0.8337, mIoU of 0.7647, recall of 0.8600, and precision of 0.8698, which outperformed other SOTA methods in all presented metrics. Our method outperformed the second performing method MSRF-Net by 7.62% in mDice, 13.1% in mIoU, 14.03% in recall, and 2.84% in precision.

**Table 7:** generalizability results of the models trained on CVC-Clinicdb and tested on Kvasir-SEG

Method	Year	mDice	mIoU	Recall	Precision
U-Net [5]	2015	0.5621	0.4050	0.4364	0.8466
U-Net++ [7]	2018	0.6783	0.5494	0.7311	0.6885
HRNetV2-W18-Smallv2 [44]	2020	0.2107	0.1363	0.2038	0.3347
HRNetV2-W48 [44]	2020	0.2349	0.2461	0.3372	0.1523
MSRF-Net [41]	2021	0.7575	0.6337	0.7197	0.8414
EG-TransUNet	**–**	**0.8337**	**0.7647**	**0.8600**	**0.8698**

### Ablation study

**Fig. 10:**
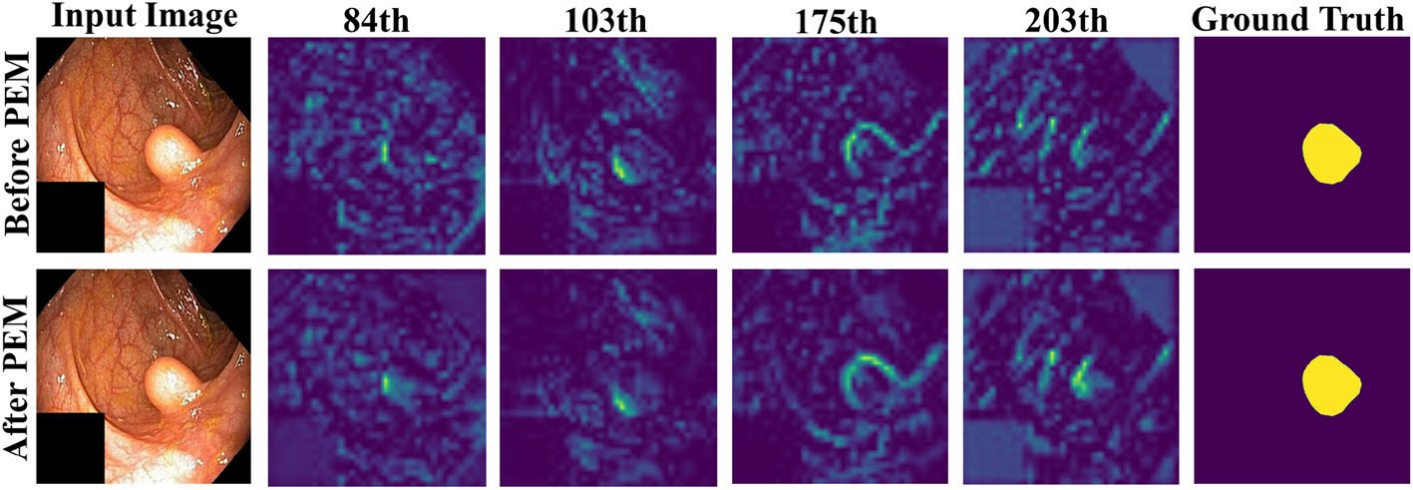
Qualitative results of PEM

**Fig. 11:**
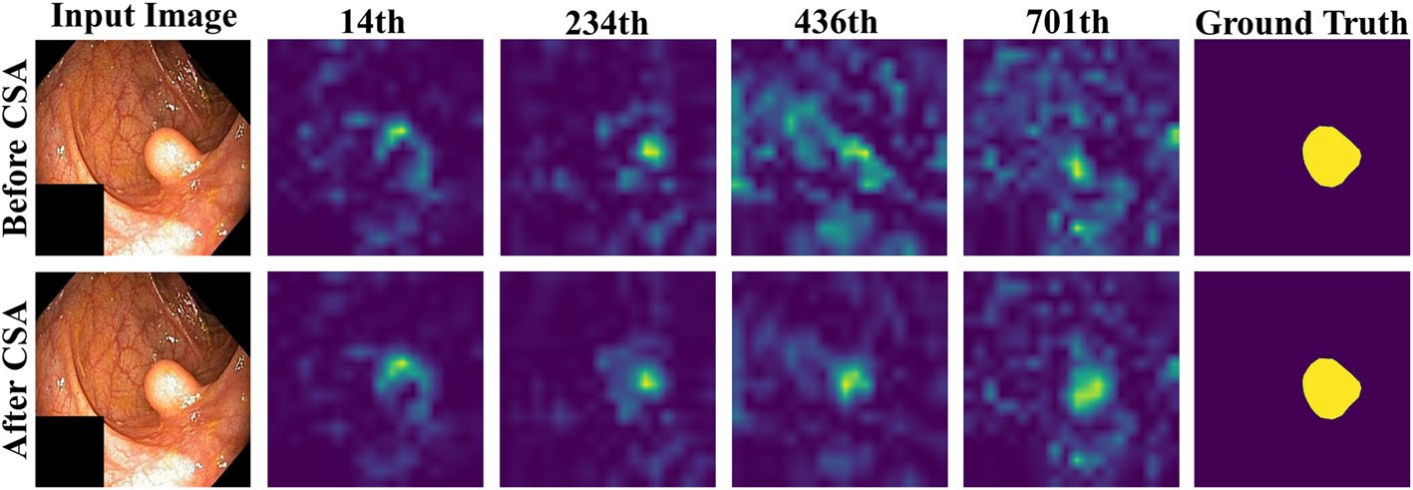
Qualitative results of CSA

**Fig. 12:**
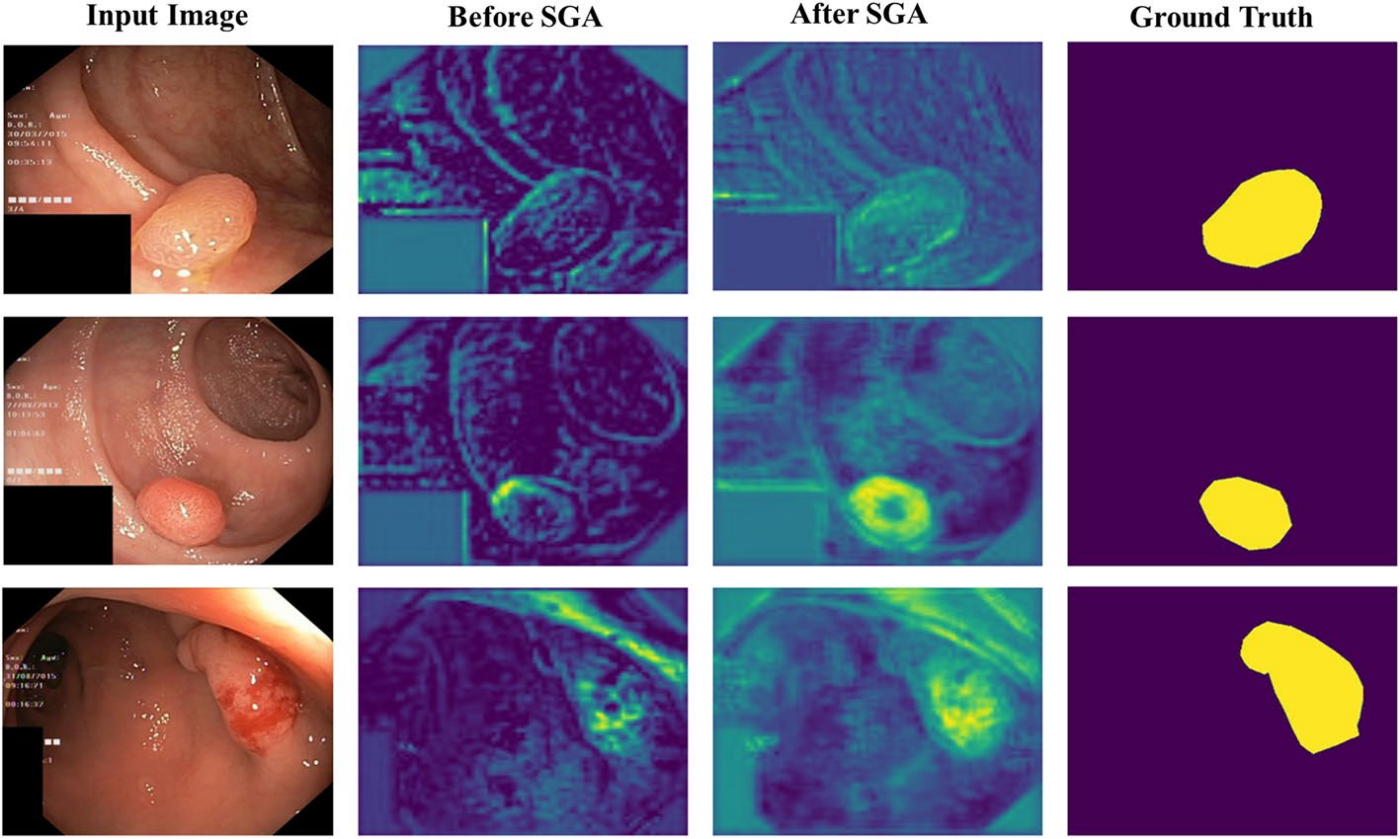
Qualitative results of SGA

We conducted an ablation study on the Kvasir-SEG data set to demonstrate the effects of PEM, semantic guided attention, and CSA with floating-point calculations. The related quantitative results are shown in Table 8 and the qualitative results are shown in Figs. 10, 11, and 12.

**Table 8:** Ablation study of EG-Transunet on the Kvasir-SEG

Method	mDice	mIoU	Recall	Precision	Flops
EG-N w/o PEM + CSA + SGA	0.8820	0.8269	0.8956	0.8990	16.3G
EG-N w/o PEM + SGA	0.9248	0.8796	0.9342	0.9368	16.9G
PAS w/o PEM + CSA	0.9227	0.8790	0.9258	0.9336	21.8G
EG-N w/o SGA + CSA	0.9263	0.8815	0.9303	0.9345	18.8G
EG-N w/o PEM	0.9284	0.8754	0.9388	0.9281	22.2G
EG-N w/o SGA	0.9301	0.8867	0.9363	0.9404	19.3G
EG-N w/o CSA	0.9334	0.8849	0.9369	0.9353	24.4G
EG-TransUNet	**0.9344**	**0.8927**	**0.9401**	**0.9436**	24.9G

For each column, the best results are highlighted

The U-shaped network was the benchmark network used in this paper, which is represented as “EG-N W/O PEM + CSA + SGA,” while “EG-N” is considered as the “full” model. The results of “EG-N w/o PEM + SGA,” “EG-N w/o PEM + CSA,” and “EG-N w/o SGA + CSA” indicated that the proposed PEM, CSA, and SGA modules could improve the segmentation quality, with almost equally effectiveness. In terms of Flops in Table 8, EG-TransUNet has only half more computation than the baseline, with no differences regarding the point of computation and magnitude, while greatly improving the segmentation performance.

The spatial feature before and after the PEM were visualized for some channels, and the 84th, 103rd, 175th, and 203rd channels were randomly selected, as shown in Fig. 10. By comparing the above and below, it can be seen that PEM could remove and undermine irrelevant texture information and enhance relevant texture information, confirming the effectiveness of PEM in enhancing feature expression and improving feature discrimination.

The 14th, 234th, 436th, and 701st channels were randomly selected to visualize the feature before and after CSA, as shown in Fig. 11. An updown comparison showed that CSA could optimize the semantic feature and improve the accuracy of semantic location information of the input medical images.

Some images of the Kvasir-SEG dataset were selected for SGA visualization analysis. As shown in Fig. 12, the overall contrast of the feature map decreased after SGA, but the value of the target region increased, thus providing more important texture information in the feature fusion stage. This in turn confirmed the effectiveness of SGA in reducing semantic gap and promoting the effect of feature fusion.

We also performed additional ablation studies by removing single module to further verify the effectiveness of our work. Compared to EG-TransUNet, the evaluation scores of “EG-N w/o PEM,” “EG-N w/o SGA,” and “EG-N w/o CSA” were reduced to varying degrees, demonstrating the effectiveness and necessity of each module. Our experimental results clearly show that all three modules could enhance each other and jointly improve the segmentation effect.

### Visualizations of the decoder stages

**Fig. 13:**
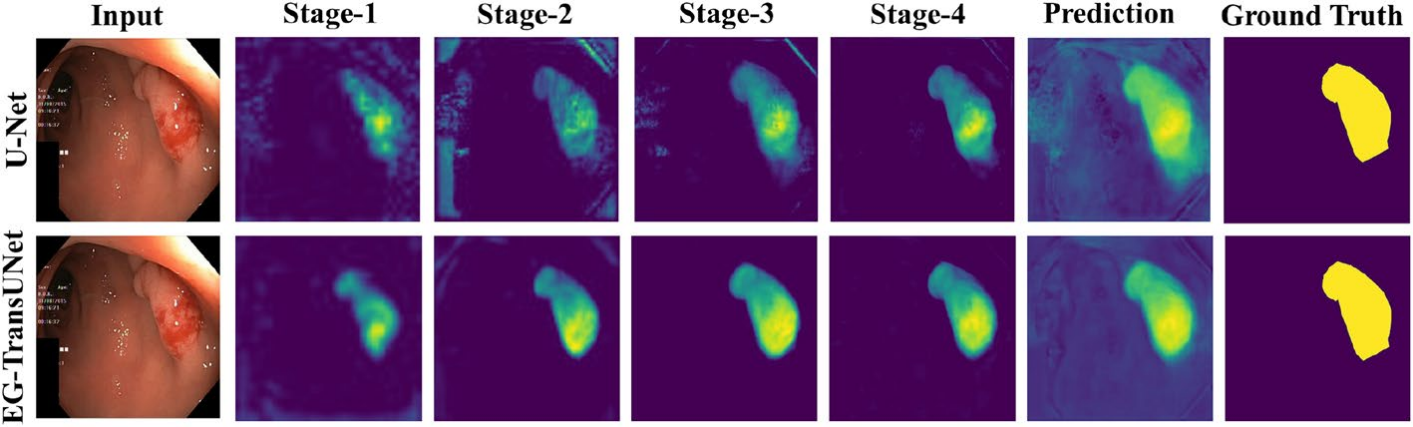
Visualizations of feature maps produced by vanilla U-Net and the proposed EG-TransUNet in different decoder stages based on the Kvasir-SEG dataset

Compared with vanilla U-Net, the proposed EG-TransUNet benefits greatly from the long-range feature dependencies and global contextual information. To further verify the ability of the proposed EG-TransUNet, we visualized feature maps from each decoder stage for both U-Net and EG-TransUNet, as illustrated in Fig. 13.

The following observations can be made based on the comparative results: (1) With the deepening of the decoding process, the high-level semantic feature and the low-level texture feature could be combined and gradually improve the edge part. In addition, the resolution of the decoded image was gradually improved, and the details of the image were gradually enriched, rendering the decoded image closer to the real result. (2) When PEM and CSA were used, the comparison of the decoding images in the first stage clearly suggests that the method in this chapter provided clearer location information and edge information compared with vanilla U-Net. (3) Although the decoding image of vanilla U-Net is gradually improved and clear, the fuzzy edge and unclearness were still unsolved. This finding underlines that the spatial feature was not fully learned to provide a discriminative edge of the lesion area and did not exploit the spatial texture details during the feature fusion. By comparison, the edge of EG-TransUNet was clear and the decoding effect was better due to SGA.

We believe that the application of EG-TransUNet architecture should not only be limited to biomedical image segmentation, but also be extended to natural image segmentation and other pixel-level classification tasks; however, further detailed validations will be necessary.

## Conclusion

In this paper, we propose a novel U-Net variant with Transformer, called EG-TransUNet, which implements the PEM, the feature fusion module based on semantic guidance attention, and the CSA module into U-Net simultaneously, and can thus greatly improve the segmentation quality of biomedical images.

In particular, PEM could enhance information with a stronger representation of the target location, optimize the inference process of fuzzy edge information, and improve feature discrimination effectively.

Meanwhile, SGA could explore and exploit the relationship between semantic and spatial texture information, eliminate the semantic gap, and realize an effective fusion of spatial texture and semantic information.

In addition, CSA could effectively capture the long-range contextual information in channel and spatial levels by using the self-attention mechanism, which improves the representation ability of the semantic feature and the accuracy of semantic location information.

Compared with previous advanced works, the proposed EG-TransUNet greatly benefits from the long-range feature dependencies of the transformer, ensuring the discriminative representations of the spatial feature, the accuracy of semantic location information, and efficient feature fusion. Consequently, we can effectively mitigate problems occurring when using the traditional U-shape architecture and obtain a competitive segmentation and generalization performance. In clinical practice, the network proposed in this paper has the ability to extract reliable discriminative features and fuse spatial and semantic information. At the same time, it can reduce various noise interference in medical image data and provide reliable high-precision medical image segmentation results that can significantly improve diagnostic accuracy.

## Data Availability

All datasets used in this paper are publicly available. The Kvasir-SEG is publicly available at https://datasets.simula.no//kvasir-seg/#download. The CVC-ClinicDB is publicly available at https://polyp.grand-challenge.org/CVCClinicDB/. The 2018 Data Science Bowl is publicly available at https://www.kaggle.com/competitions/data-science-bowl-2018/data. The ISIC -2018 Challenge is publicly available at https://challenge.isic-archive.com/data/#2018. The GLAS is publicly available at https://warwick.ac.uk/fac/cross_fac/tia/data/glascontest/download/.
